# Utilizing Consumer Health Informatics to Support Management of Hypertension by Clinical Pharmacists in Primary Care: Study Protocol

**DOI:** 10.2196/resprot.8059

**Published:** 2017-10-10

**Authors:** Lorraine R Buis, Dana N Roberson, Reema Kadri, Nicole G Rockey, Melissa A Plegue, Hae Mi Choe, Caroline R Richardson

**Affiliations:** ^1^ Department of Family Medicine University of Michigan Ann Arbor, MI United States; ^2^ University of Michigan Medical Group Pharmacy Innovations and Partnerships Ann Arbor, MI United States

**Keywords:** hypertension, medication adherence, cell phones, telemedicine

## Abstract

**Background:**

Hypertension (HTN) is a major public health concern in the United States given its wide prevalence, high cost, and poor rates of control. Multiple strategies to counter this growing epidemic have been studied, and home blood pressure (BP) monitoring, mobile health (mHealth) interventions, and referrals to clinical pharmacists for BP management have all shown potential to be effective intervention strategies.

**Objective:**

The purpose of this study is to establish feasibility and acceptability of BPTrack, a clinical pharmacist-led mHealth intervention that aims to improve BP control by supporting home BP monitoring and medication adherence among patients with uncontrolled HTN. BPTrack is an intervention that makes home-monitored BP data available to clinical pharmacists for use in HTN management. Secondarily, this study seeks to understand barriers to adoption of this intervention, as well as points of improvement among key stakeholders, so that larger scale dissemination of the intervention may be achieved and more rigorous research can be conducted.

**Methods:**

This study is recruiting up to 25 individuals who have poorly controlled HTN from a Family Medicine clinic affiliated with a large Midwestern academic medical center. Patient participants complete a baseline visit, including installation and instructions on how to use BPTrack. Patient participants are then asked to follow the BP monitoring protocol for a period of 12 weeks, and subsequently complete a follow-up visit at the conclusion of the study period.

**Results:**

The recruitment period for the pilot study began in November 2016, and data collection is expected to conclude in late-2017.

**Conclusions:**

This pilot study seeks to document the feasibility and acceptability of a clinical pharmacist-led mHealth approach to managing HTN within a primary care setting. Through our 12-week pilot study, we expect to lend support for this approach, and lay the foundation for translating this approach into wider-scale implementation. This mHealth intervention seeks to leverage the multidisciplinary care team already in place within primary care, and to improve health outcomes for patients with uncontrolled HTN.

**Trial Registration:**

Clinicaltrials.gov NCT02898584; https://clinicaltrials.gov/ct2/show/NCT02898584 (Archived by WebCite® at http://www.webcitation.org/6u3wTGbe6)

## Introduction

### Background and Significance

Hypertension (HTN) is a serious public health concern in the United States, affecting approximately 78 million adults and burdening the health care system with an estimated US $42.9 billion in direct costs in 2010 [1,2]. HTN is a key risk factor for heart disease and stroke, which are the first and fourth leading causes of death in the United States, respectively [3]. Of patients with HTN, it is estimated that only approximately 50% achieve blood pressure (BP) control, and approximately 20% remain unaware of their condition [1]. Given the prevalence and high cost of HTN, coupled with poor rates of control, identifying strategies to assist patients in managing their HTN is imperative.

An important and effective strategy for HTN management is home BP monitoring [4-7], yet problems with data quality and latency can be abundant when patients maintain paper-based logs for self-monitoring [8,9], potentially compromising the effective and efficient use of paper-based logs in primary care. Additionally, clinical pharmacists can help manage chronic conditions such as HTN in primary care settings [10,11] and have themselves been shown to be an effective intervention for patient HTN management [12,13]. Despite evidence suggesting the efficacy of home BP monitoring and clinical pharmacists for HTN management, neither strategy is being widely used in primary care settings.

One potential solution that shows great promise is the use of mobile health (mHealth) approaches to bridge the gap between home monitoring and clinical pharmacist care. Approximately 95% of American adults have a cell phone, 77% have a smartphone, and the rate of smarphone adoption is increasing rapidly [14]. Moreover, the majority of individuals living with one or two chronic conditions report tracking at least one health indicator (70% and 80% respectively); a task that is greatly supported through the use of mobile technology [15].

mHealth interventions may therefore be a viable strategy to close the loop between patient self-monitoring and clinician management, thus potentially improving HTN management. The current utility of such interventions is often reduced however, as they are typically patient-facing and do not support bidirectional patient-provider communication. However, through a bidirectional intervention in which patient-provider interaction and automatic transmission of electronic data from home BP monitors to clinicians can occur in real-time, mHealth interventions may be a practical solution. Efficiencies in patient management could potentially lead to increases in the number of hypertensive patients that a clinical pharmacist could manage, as well as improvements in the quality of patient BP management. Moreover, mHealth interventions often contain other features that have been linked to chronic disease self-management, including data tracking, general/tailored education, and reminders, all of which may contribute to improved HTN control [16,17].

### Study Objective

The purpose of this study is to establish feasibility and acceptability of BPTrack, which is a clinical pharmacist-led mHealth intervention that supports home BP monitoring and medication adherence among patients with uncontrolled HTN, and makes collected data available to the pharmacist for use in HTN management. Secondarily, this study seeks to understand barriers to adoption, as well as points of improvement among key stakeholders, so that larger scale dissemination of the intervention may be achieved, and more rigorous research can be conducted.

## Methods

### Overview

We are currently conducting a 12-week pre/postintervention pilot study of BPTrack, a clinical pharmacist-led mHealth intervention, which seeks to improve BP control among uncontrolled hypertensive patients recruited from a primary care setting, and who are receiving treatment for their HTN under the care of a clinical pharmacist embedded in the primary care clinic. The final protocol closely mirrored what was originally proposed to the funder (see Multimedia Appendix 1 and Multimedia Appendix 2 for original review scoring forms). The methods for this study have been approved by the University of Michigan IRBMED Institutional Review Board (HUM00105772).

### BPTrack Intervention Description

The BPTrack intervention supports patient home BP monitoring and medication adherence tracking for sharing with a clinical pharmacist. BPTrack consists of two different mobile apps developed by the Tactio Health Group and modified from their TactioRPM Platform; one for the patient, and one for the clinical pharmacist. These modified mobile apps are intended for our research purposes only, have been rebranded with appropriate program logos, and are distributed by the University of Michigan via the Apple and Google Play app stores.

**Figure 1:**
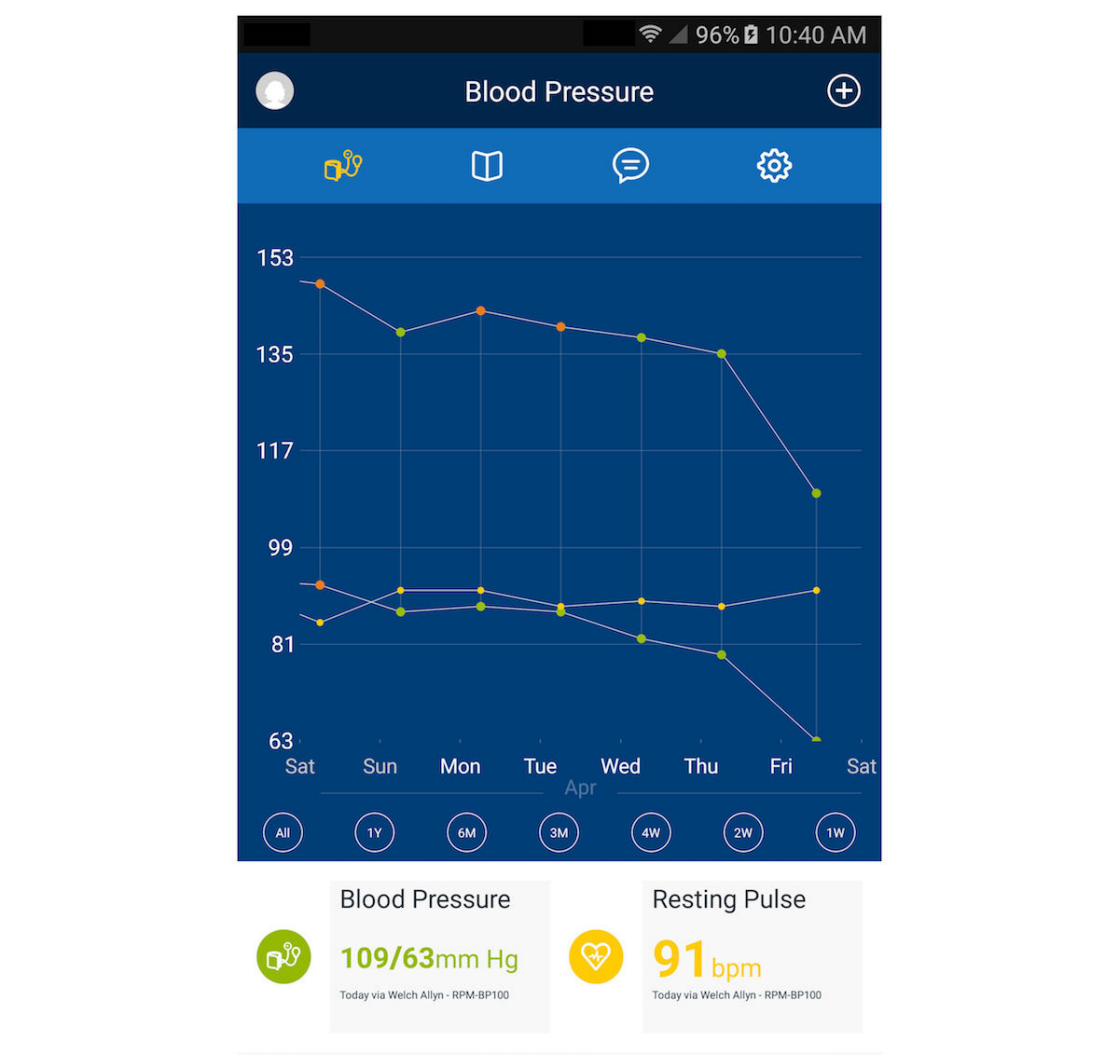
BPTrack blood pressure graph screen.

**Figure 2:**
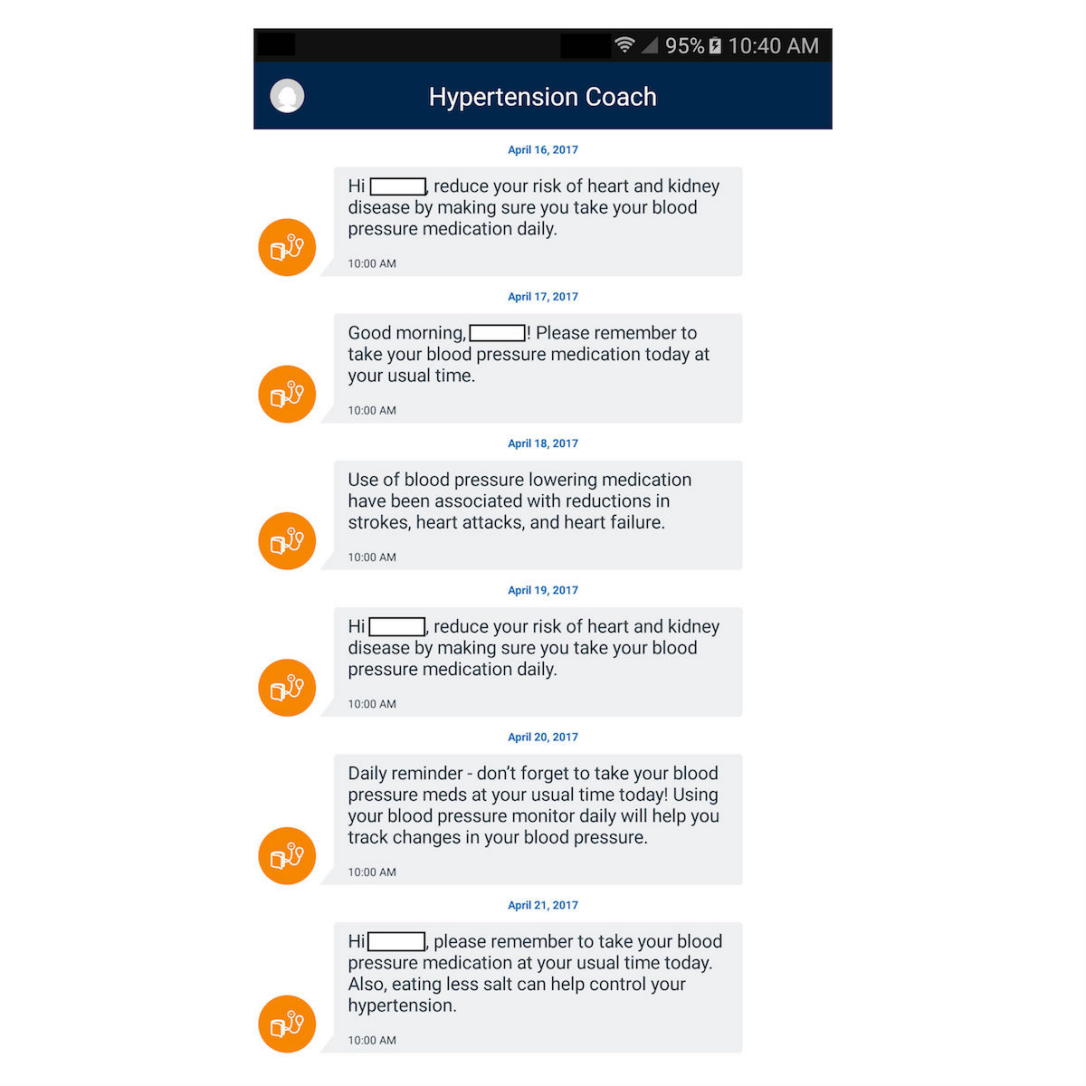
BPTrack medication reminder screen.

BPTrack is a patient-facing smartphone app for iOS and Android that supports home BP monitoring, medication adherence, and communication with a clinical pharmacist who is managing patients’ HTN. BPTrack allows people to measure their BP using a compatible Bluetooth-enabled BP cuff (this study provides patient participants with a Welch Allyn Remote Monitoring Upper Arm Blood Pressure Device RPM-BP100), after which the BP reading is automatically synced with the BPTrack smartphone app, and transmitted to a secured cloud server. Individuals can also manually enter their BP data into the app, without syncing the BP cuff. Within the visual display, patient participants are provided a graph of their BP data over time (Figure 1), as well as mean, minimum, and maximum values for systolic BP (SBP), diastolic BP (DBP), and pulse. Color-coded data points on the graph highlight how a BP value compares to BP target goals; two hues of green represent normal BP or prehypertension, orange represents stage 1 HTN, and red represents stage 2 HTN (refer to Table 1 for actual assigned SBP and DBP ranges within BPTrack). To self-monitor medication adherence, BPTrack sends daily medication reminders that are coupled with educational messages focused on the importance of medication adherence for managing HTN (Figure 2). In addition, users can record their adherence data in free text via a *Notes* feature. Finally, BPTrack provides a mechanism to send in-app messages directly to the clinical pharmacist.

**Figure 3:**
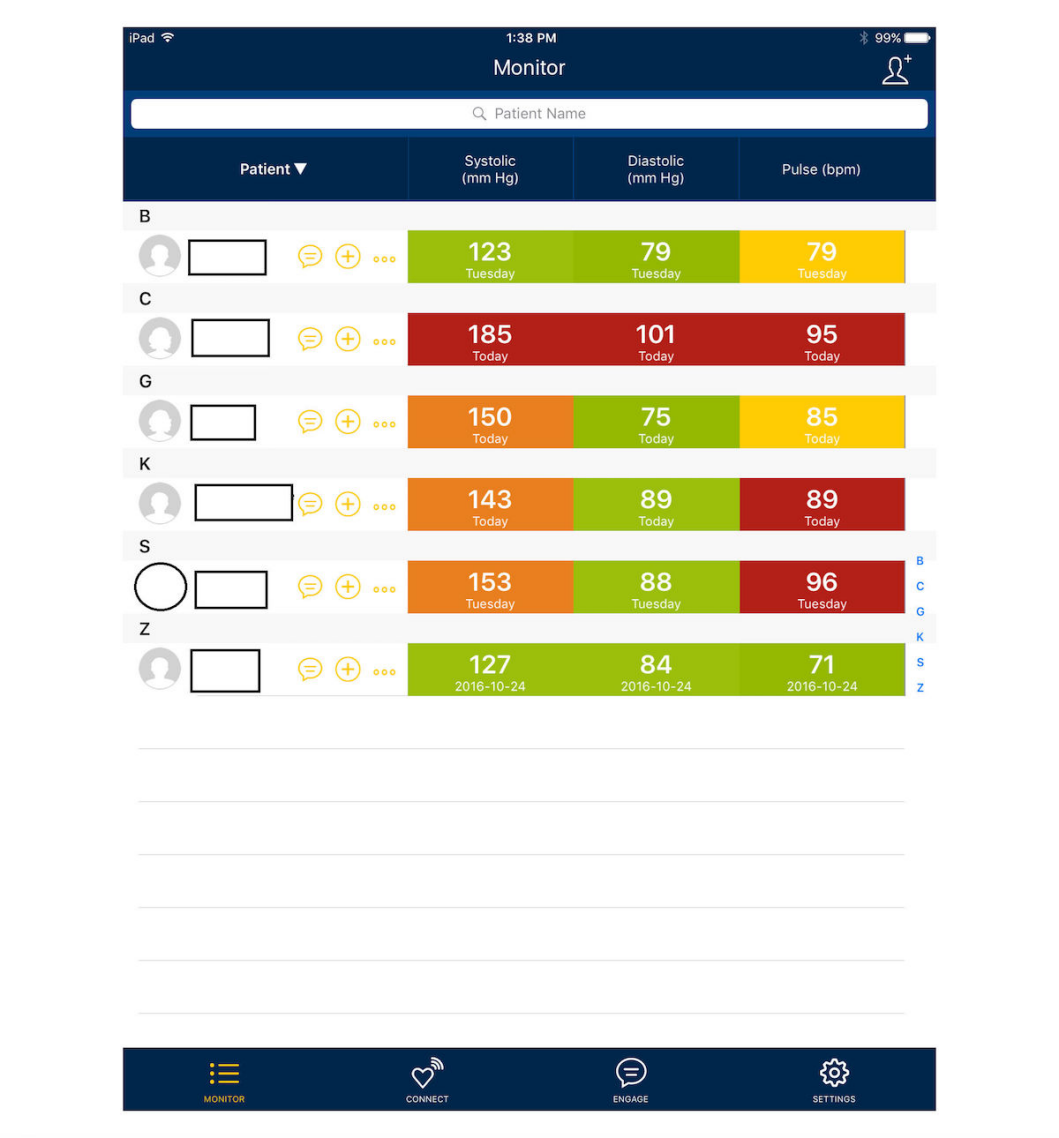
BPTrack Pharm main screen.

**Figure 4:**
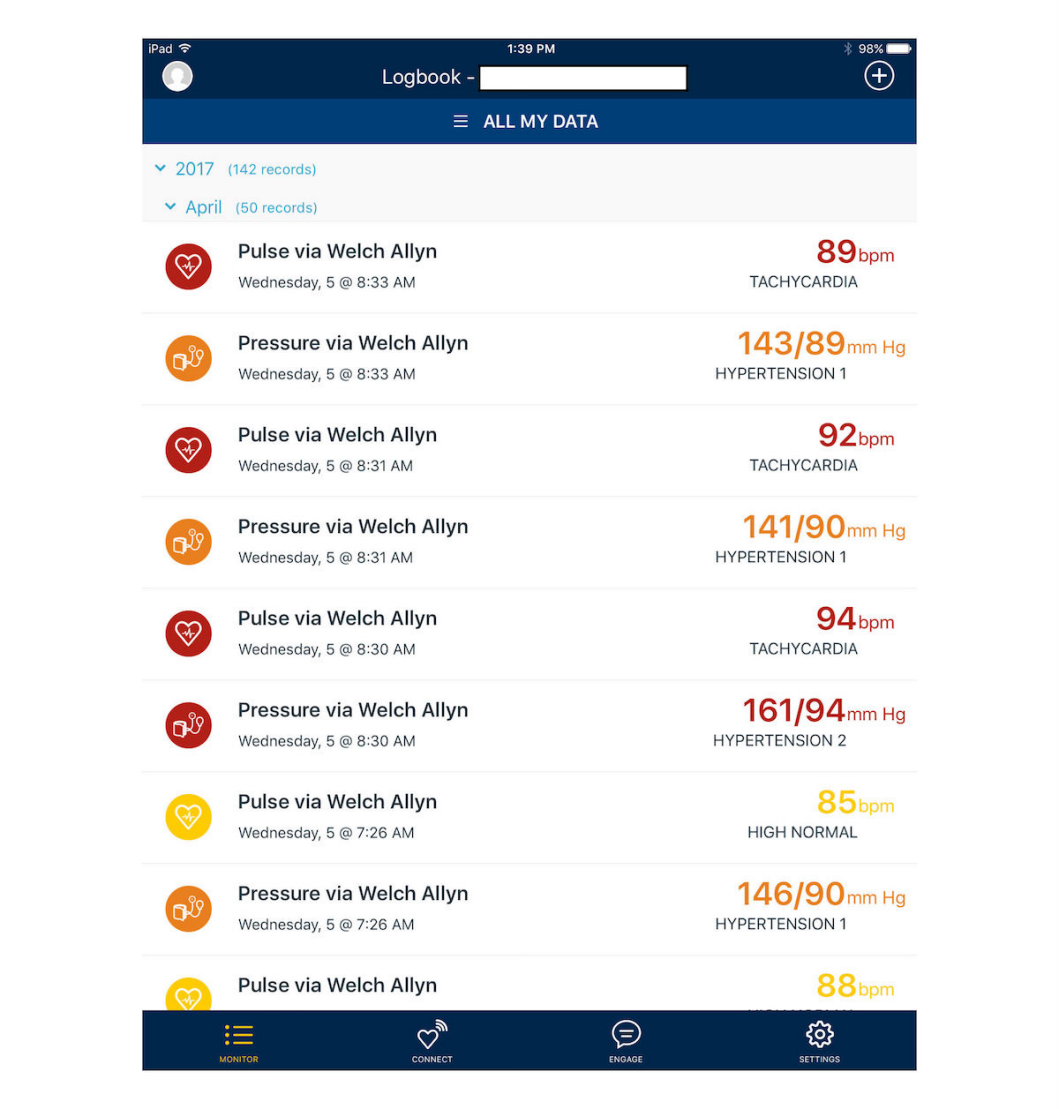
BPTrack Pharm individual screen.

BPTrack Pharm is a mobile app for the iPad, which is used by clinicians to monitor collected BP and medication adherence data from patients using the BPTrack app. BPTrack Pharm presents clinicians with a color-coded dashboard view to quickly highlight all enrolled patients’ BP control (Figure 3), as well as a more detailed view for each individual patient, which allows the clinical pharmacist to view all collected BP and adherence data for each enrolled patient (Figure 4). BPTrack affords clinicians the ability to set custom BP goals for individuals, and to directly message a patient via the app.

**Table 1:** Assigned SBP and DBP ranges for blood pressure categorization in BPTrack.

Category	Color	Systolic Blood Pressure	Diastolic Blood Pressure
Optimal	Green	<120 mmHg	<80 mmHg
Prehypertension	Light green	≥120 mmHg to <140 mmHg	≥80 mmHg to <90 mmHg
Stage 1 Hypertension	Orange	≥140 mmHg to <160 mmHg	≥90 mmHg to <100 mmHg
Stage 2 Hypertension	Red	≥160 mmHg	≥100 mmHg

### BPTrack Theoretical Underpinnings

Our approach to HTN management, which relies on daily BP and medication adherence self-monitoring, is rooted in a self-regulatory framework. In the theory of self-regulation, individuals participate in self-directed behaviors, thought to be managed through a dynamic feedback loop. In this loop, individuals process information about their past behavior and integrate the information into goals and motivation to change future behaviors (ie, self- monitoring) [18]. By engaging in daily BP and medication adherence self-monitoring, patient participants will have the opportunity to see progress made toward meeting BP goals, which (when combined with self-reflection on behaviors) should tap into the dynamic feedback loop to lead to possible behavior change.

### Setting

This pilot study is being conducted with patients at a Family Medicine clinic affiliated with a large Midwestern academic medical center. The participating site generates over 29,000 patient visits annually (72% of which are adult visits) and serves a predominantly blue-collar, underserved African-American and Hispanic population. HTN is a prevalent chronic condition that is treated at the recruiting clinic.

### Sample

We are enrolling up to 25 patient participants for this pilot study. To be eligible for participation, enrollees must be ≥18 years of age, have a smartphone compatible with the mobile intervention, have been previously diagnosed with HTN, have uncontrolled HTN (SBP >140 mmHg and/or DBP >90 mmHg on repeat measurements), be under the care of a primary care physician at the recruiting clinic, be taking at least one antihypertensive medication, and be English-speaking.

Potential enrollees are excluded if they are >65 years of age, are already under the care of a clinical pharmacist for HTN management or under the care of a cardiologist, are pregnant, have serious existing medical conditions that may make BP control difficult or necessitate frequent hospitalization (such as previous diagnosis of resistant HTN, steroid dependent asthma or emphysema, cirrhosis or hepatic failure, stage C or D chronic heart failure, stage IV or V chronic kidney disease, and terminal cancer or ongoing active chemotherapeutic or radiation therapy), or have other serious medical conditions (eg, stroke, dementia) that may affect their ability to self-monitor their BP.

### Recruitment

Patient participants are recruited through recruitment flyer dissemination by clinic staff (physicians, medical assistants, clinical pharmacist, or other clinic staff) to potentially eligible participants, as well as targeted recruitment letters. To facilitate provider and staff referral, study flyers were provided to clinic staff and providers so that they can be handed directly to potentially eligible individuals as they are seen in clinic. Moreover, study staff are providing primary care physicians with lists of potentially eligible patients, identified through the electronic medical records system, who are scheduled to be seen in the clinic. The clinical pharmacist on this study is also directly recruiting patients who meet eligibility criteria. Recruitment flyers list a study hotline that individuals must call in order to be screened for participation in the study.

In order to send targeted recruitment letters, study staff are conducting automated pulls of patient data from electronic health records to obtain mailing lists of patients at the recruiting site who meet clinical eligibility criteria. These targeted recruitment letters inform recipients that they are potentially eligible for a research study focused on HTN management and that they may receive a phone call to solicit their participation. In addition, recruitment letters provide contact information for study staff so that interested individuals can directly contact the team. Shortly after recruitment letters are distributed, study staff are calling potentially eligible participants to solicit participation.

### Procedures

Any individual patient at the recruiting site who expresses interest by calling the study hotline is subsequently screened by phone. Once screened as eligible, patient participants are scheduled for an informed consent and baseline data collection appointment with study staff at the recruitment site, or another affiliated space. These visits take approximately 90 minutes to complete.

After baseline data collection is complete, study staff help patient participants download the intervention app onto their smartphone, and train them on how to use the app. Participants are also given a Bluetooth-enabled BP monitor to take home, and are instructed on how to use it, as well as how to sync it to BPTrack. All BP monitors are checked before the patient participant leaves the baseline data collection visit, and are tested against a manual BP reading taken by a trained study staff member. Although we are not calibrating the home BP monitors, we do look to see how well their readings compare with the manual BP readings. Once enrolled in the study, the clinical pharmacist sees patient participants as needed for ongoing HTN management.

Patient participants are also instructed on the BP monitoring protocol that we will adhere to in this study, and are instructed to take three consecutive BP readings twice daily, at varying times across days. Prior to BP monitoring, patient participants are instructed to: go to the bathroom before taking their BP, if needed; keep their arm at heart level by resting it on a table during monitoring; sit in a chair with a back, with feet flat on the floor for at least five minutes prior to monitoring; avoid tobacco, caffeine, or alcohol for 30 minutes prior to BP monitoring; avoid taking their BP immediately after exercise, when emotionally upset, or in pain; and to avoid talking while taking their BP.

In the event that a patient participant has an SBP >160 mmHg, SBP <100 mmHg, DBP >100 mmHg, or DBP <60 mmHg, they are instructed to wait five minutes and then recheck their BP using the previously described protocol. Patient participants are also instructed that if BP readings exceed these critical thresholds for three days in a row, they should contact the clinic. Finally, patient participants are instructed to call the clinic immediately if they are experiencing symptoms of dizziness, chest pain, severe headache, or visual changes.

Patient participants are instructed to follow the home BP monitoring protocol for 12 weeks, to ensure that the BP readings sync with the BPTrack app, and to self-monitor BP medication adherence. During this 12-week intervention period, the project’s clinical pharmacist monitors home BP and medication adherence readings through the BPTrack Pharm clinician app, and manages patient participant HTN in concordance with their normal clinical practice. In the event that a patient participant exhibits continued elevated BP readings, the clinical pharmacist contacts them to determine whether the patient needs to come in to the clinic for follow-up, or whether to continue to monitor through the app. The clinical pharmacist is exercising their best clinical judgment in managing the research participants’ BP.

At the conclusion of the 12-week intervention period, patient participants meet with study staff for their 12-week follow-up data collection visit, which takes approximately 60 minutes to complete. At this visit, the patient participants complete the Adherence to Refills and Medications Scale (ARMS) [19], they are asked to bring in all antihypertensive medications for a pill count, and interviews are audio recorded.

To compensate patient participants for their participation in the study and to reduce attrition, individuals receive US $25 upon completion of their 12-week follow-up data collection visit. Patient participants are also allowed to keep the BP monitor used in the study (valued at approximately US $100/monitor). In addition to incentives offered for participation, additional retention strategies implemented in past and current projects are being used, such as routinely obtaining contact information for the participant and up to three friends/relatives who can help locate them, as well as baseline and follow-up appointment reminders via phone and/or email. Individuals who leave the study early are compensated on a pro-rated basis, and are required to return the BP monitor.

After the study has finished, we will conduct interviews with the study’s clinical pharmacist and other key stakeholders at the recruiting clinical site. Key stakeholders will be recruited through direct solicitation via email and phone calls; these stakeholders are expected to include clinic medical directors, primary care physicians, health systems administrators, and information technology personnel. These interviews will be audio recorded and transcribed for further analysis.

### Measures

Through this study, we are collecting a variety of different measures to help determine the feasibility and acceptability of utilizing this approach in a primary care setting, including: *Patient Participant Characteristics*, *Blood Pressure*, and *Medication Adherence*.

#### Patient Participant Characteristics

At baseline, we administer an investigator-developed survey to assess patient participant demographics, health status, HTN history, medication use, and other characteristics.

#### Blood Pressure

At both baseline and 12-week follow-up, patient participant BP is assessed in the clinic by a manual BP reading taken by a trained study staff member or clinic staff. In addition, home BP readings will be downloaded from the BPTrack Secured Cloud at the conclusion of the study for further analysis.

#### Medication Adherence

At baseline and the 12-week follow-up, we assess medication adherence with two different measures: the ARMS and pill counts. The ARMS is a 14-item instrument that consists of two sub-scales that assess taking medications as prescribed, and refilling medications on schedule. The ARMS has been validated for use in a population with chronic disease and has been shown to have good performance characteristics among individuals with low literacy levels [20]. Upon enrolling in the pilot study, all patient participants are instructed to save all pill bottles for antihypertensive medications, even if empty, and bring them to the follow-up assessment. Patient participant HTN medications are identified from the patient’s medical record. Information required for the pill count calculation is recorded from each prescription label including: drug name, strength, and dosage form; instructions for use; quantity dispensed; and dispensing date. Pill count adherence is determined by the following formula, [19]:

% adherence = (quantity dispensed - quantity remaining)/([prescribed # tablets/day] x [# days between dispensing date and interview]) x 100

### BPTrack and BPTrack Pharm Utilization

At the conclusion of the study, BPTrack and BPTrack Pharm utilization for patient and clinical pharmacist participants will be documented through log file analysis, with specific focus on the number of patient participant home BP readings, as well as the frequency and duration of participant use of the BPTrack and BPTrack Pharm apps.

### Health Care Utilization

Patient participant health care utilization will be conducted through a retrospective chart review of enrolled individuals in order to document the number and reason for primary care visits during the course of this study, including the frequency and nature of contacts with the clinical pharmacist (phone or in-person), with a primary care physician at the recruiting clinic, and the Emergency Department at the academic medical center.

### Patient Participant Perceptions

At the conclusion of the 12-week study period, we will conduct an investigator-developed survey focused on patient participant perceptions of BPTrack including ease of use, usefulness, impact on health outcomes, and satisfaction. We will also conduct an open-ended interview using a standardized script to elicit additional qualitative feedback.

### Key Stakeholder Utilization and Perceptions

At the conclusion of this pilot study, we will conduct an in-depth interview with the clinical pharmacist involved in this study to better understand perceptions of the system from a clinical perspective. Moreover, throughout the course of the pilot study, the pharmacist has been asked to maintain detailed logs of the time spent using the app, time spent monitoring and following-up with patient participants, and perceptions of the program. These logs will be collected and analyzed in order to document the personnel needs to maintain such a program. We will also conduct key stakeholder interviews with clinic medical directors, primary care physicians, health system administrators, and information technology personnel at the recruiting site and other affiliated clinical sites, to identify potential barriers to broader intervention dissemination, as well as strategies to overcome those challenges.

### Statistical Analysis Plan

Descriptive statistics will be used to describe patient participant characteristics and perceptions, mHealth utilization, and clinical pharmacist utilization and perceptions. Categorical data will be displayed as frequencies and percentages, and Chi-square tests will be used for comparisons. SBP and DBP will be expressed as mean (standard deviation) and pre/postintervention BP means will be compared using 2-tailed paired samples t-tests.

The effect of clinical pharmacist contact with patient participants will be evaluated through changes in utilization of the mHealth app and changes in BP levels surrounding the time of contact. Models will be used to separately evaluate SBP and DBP levels over time, including pharmacist contact as a time-varying covariate using a mixed-effects regression approach. The mixed-model analysis accounts for the correlation of BP measures on the same individual across time. Similar analyses will be carried out on daily utilization of the mHealth app. Daily usage will be coded as a binary response and modeled using generalized estimating equations, including pharmacist contact with patient participants as a covariate to identify the odds of app usage before and after contact.

## Results

Patient participant recruitment for the pilot study began in November 2016, and data collection is expected to conclude in late-2017.

## Discussion

### Principal Results

At the conclusion of this study, we expect to be able to demonstrate the feasibility and acceptability of using an mHealth approach for supporting clinical pharmacist-led HTN management in a primary care clinic. By placing clinically relevant home BP and medication adherence data from patients directly into the hands of a medical professional who has the potential to act upon it in a clinically relevant and timely manner, we may be better able to improve patient care for individuals who struggle to manage HTN. Moreover, by incorporating mobile devices that are already used by the target population, we are taking an inherently patient-centered approach to HTN management. This “bring-your-own-device” approach ensures that we are asking people to utilize technologies already incorporated into daily life, which is necessary for adequate translation from bench to bedside. We anticipate that through this pilot study, we may be able to document efficiencies that this type of program may create, and learn from our stakeholders how to overcome barriers to implementation and adoption, in advance of a larger scale roll-out.

### Limitations

Perhaps the largest limitation of this pilot study is our limited sample size, which was chosen due to the pragmatics of recruitment and budgetary constraints. As highlighted by Leon et al, “the primary role of a pilot study is to example the feasibility of a research endeavor” [21]. Our intended sample size is large enough to satisfy the rule of thumb of 12 participants per group (set forth by Julious et al [22]) as appropriate for guiding sample size selection for pilot studies. Although we may not have adequate power to detect effects, this is a true feasibility trial in which we will be able to obtain preliminary data to support and plan for future implementations. Feasibility will be assessed on many different levels, including the feasibility of using BPTrack amongst our target population, within the primary care setting, and within the health system. In addition, the lack of a control or other comparison group is a limitation of our study design; however, given that this is a pilot study to preliminarily investigate the use of this approach in a clinical setting, our single group design is warranted. Future work should seek to incorporate a more robust research design.

### Comparison to Prior Work

This type of clinical pharmacist-led mHealth approach to improving HTN management has been demonstrated to be effective within research settings. Although it was undertaken in a different population, a recent study by Margolis et al found that 57.2% of participants randomized to receive an intervention that involved pharmacist case management for HTN, plus home BP telemonitoring, were able to bring their BP down to controlled levels at 6 and 12 months, versus 30.0% of those receiving usual care [13]. Moreover, at 6-months postintervention, 71.8% of intervention participants had controlled BPs versus 57.1% in usual care [13]. These findings provide strong evidence in support of this approach, yet their study was limited to a research setting, and was not integrated into routine clinical practice. Our pilot study seeks to lay the foundation for translating the Margolis et al findings into practice [13].

### Conclusion

This pilot study was designed to document the feasibility and acceptability of a clinical pharmacist-led mHealth approach to managing HTN within a primary care setting. In our 12-week pilot study, we expect to lend support for this approach and lay the foundation for translating this approach into wider-scale implementation, in order to leverage the multidisciplinary care team already in place within primary care, and to improve health outcomes for patients with uncontrolled HTN.

## Appendix

Multimedia Appendix 1 MICR peer review report 1.

Multimedia Appendix 2 MICR peer review report 2.

## Abbreviations

ARMS: Adherence to Refills and Medications Scale
BP: blood pressure
DBP: diastolic blood pressure
HTN: hypertension
mHealth: mobile health
SBP: systolic blood pressure
