# Supplementary material for: Utilizing Consumer Health Informatics to Support Management of Hypertension by Clinical Pharmacists in Primary Care: Study Protocol
Source: JMIR Res Protoc. 2017 Oct 10;6(10):e193. doi: 10.2196/resprot.8059 (PMC5654738; doi:10.2196/resprot.8059)
Supplement: Multimedia Appendix 1 [file resprot_v6i10e193_app1.pdf]

MICHR MICHIGAN INSTITUTE FOR CLINICAL & HEALTH RESEARCH UNIVERSITY OF MICHIGAN

Research into Practice (SRC) Scoring Form – Round 18

Project Title: Utilizing Consumer Health Informatics to Support Management of Hypertension

Principal Investigator: Buis

Reviewer 1

<table>
<tr><th>Criteria</th><th>Review Questions & Comments</th><th>Score</th></tr>
<tr><td rowspan="2">Overall Impact</td><td>Overall Impact Score: The likelihood for the project to exert a sustained, powerful influence on the research field(s) involved, in consideration of the following five core review criteria, and additional review criteria (as applicable for the project proposed). Please provide a paragraph summarizing the factors that informed your Overall Impact score. (required)</td><td>3</td></tr>
<tr><td colspan="2">Strengths: This well written “pilot” application by an Assistant Professor in Family Medicine proposes to test the feasibility of (actually described as a “proof of concept” proposal) a mobile health intervention for hypertension management. Specifically, the proposal seeks to combine the use of technologies (smart phones, apps, and clinical health monitoring devices) and specific health care providers (clinical pharmacists) to 1) improve the monitoring and management of hypertension in outpatients and 2) increase the number of patients that a clinical pharmacist could manage. The efficient management of chronic diseases is a very important health challenge that this proposal will address.

Weaknesses: Weaknesses include the absence of a more detailed data analysis plan, especially of the qualitative data gathered from key stakeholders.</td></tr>
<tr><td>Significance</td><td>1.The potential for this project to address a significant health care challenge and advance mechanistic, diagnostic and /or therapeutic understanding of a clinical problem. If the aims of the project are achieved, how will scientific knowledge, technical capability, and or/clinical practice be improved? How will successful completion of the aims change the concepts, methods, technologies, treatments, services, or preventative interventions that drive this field?
Strengths and Weaknesses (required)</td><td>2</td></tr>
</table>

	Strengths: The chronic disease of hypertension and its management is a significant health challenge and the use of technology as well as the coordinated involvement of relevant health care professionals (in this case, clinical pharmacists) would be an important contribution to future health care practice and delivery. Testing the feasibility of this mobile/ pharmacist-led intervention and identify challenges to its implementation will be a major contribution to the health care field, potentially leading to improved patient outcomes and more efficient patient monitoring by health care professionals.<br><br>Weaknesses:

Research into Practice (SRC) Scoring Form – Round 18

<table>
<tr><td rowspan="2">Investigators</td><td>**2.** Are the PIs, collaborators and other researchers well suited to the project? **If Early Stage Investigators**, do they have the appropriate experience and training, and is a mentoring program with an established investigator outlined? **If the PI is a Senior Investigator** (Associate Prof. and above), has it been clearly demonstrated that the proposed work is a departure from prior research? If the project is collaborative or multi-PD(s)/PI(s), do the investigators have complementary and integrated expertise; is their leadership approach, governance and organizational structure appropriate for the project? **Strengths and Weaknesses** (required)</td><td>**2**</td></tr>
<tr><td colspan="2">Strengths: The PI is an Assistant Professor with a multidisciplinary research background and specific experience in the use of mobile interventions. She has related publications indicating ability to carry out this work. She is partnered with an Clinical Associate Professor of Pharmacy who is also the Director of Pharmacy Innovation & Clinical Practices in the University of Michigan Health System. Her mentor is a health services researcher and an Associate Professor of Family Medicine with whom she has published related work.

Weaknesses:</td></tr>
<tr><td rowspan="2">Innovation</td><td>**3.** The potential for this project to develop novel concepts, approaches, methodologies, tools or technologies in the field(s). Are the concepts, approaches or methodologies, instrumentation, or interventions novel to one field of research o novel in a broad sense? **Strengths and Weaknesses** (required)</td><td>2</td></tr>
<tr><td colspan="2">Strengths: While the concept of using mobile devices to monitor and record health data is not new, the combined collection of 1) blood pressure readings on patient smart phones directly from the sphygmomanometer with 2) medication adherence data and transmission directly to a clinical pharmacist who is monitoring 1) and 2) provides real time information that can be acted upon more immediately. This proposal will also gather data (qualitative) from other “stakeholders,” such as clinic medical directors, primary care physicians, health system administrators, and IT personnel to assist in fine tuning the intervention.</td></tr>
</table>

	Weaknesses:	
Approach	**4.** Are the overall strategy, methodology, and analyses well-reasoned and appropriate to accomplish the specific aims of the project? Are potential problems, alternative strategies, and benchmarks for success presented? For research not directly involving humans, has the PI clearly described how the next step in the overall research program will be translated into human-based, clinical research? **Strengths and Weaknesses**	**3**
	Strengths: The overall strategy presented is sound. The plan proposes to recruit 25 participants and given the training necessary for participants and staff and the timeframe presented, this seems like a realistic/achievable number. Analysis plan seemed adequate. Potential problems are discussed<br><br>Weaknesses: While a fair amount of detail was provided about the pre/post intervention data, more details of what information regarding 1) participant perceptions (a survey was proposed) and 2) qualitative data gathered from "stakeholders" would be helpful. Specifically, how will these data be relate to the specific aims and the stated "anticipated problems?"	
Environ ment	**5.** Will the scientific environment in which the work will be done contribute to the probability of success? Are the institutional support, equipment and other physical resources available to the investigators adequate for the project proposed? Will the project benefit from unique features of the scientific environment, subject populations, or collaborative arrangements? Is the proposed budget and available resources fully justified and adequate to complete the work in the proposed **1 year time period**? **Strengths and Weaknesses** (required)	**2**

Research into Practice (SRC) Scoring Form – Round 18

<table>
<tr><td rowspan="1"></td><td colspan="2">Strengths: This research team has collaborated with related experts in the past, based on past publications. They appear to have chosen and are familiar with appropriate software for the proposed study. The proposed budget appears justified with a fair amount of in-kind personnel time noted.

Weaknesses:</td></tr>
<tr><td rowspan="2">Program</td><td>6. Overall, does the application meet the objectives of the specific RFA and/or goals of the Pilot Grant Program? Including, but not limited to:
➢ To assist early career investigators by providing funding support that will enable them to establish a clinical & translational research path.
➢ To assist established basic science investigators to move their research into the translational research arena.
➢ To support clinicians interested in pursuing innovative research questions in the clinical setting or in the community.
➢ The likelihood that this proposal will lead to external funding.</td><td>2</td></tr>
<tr><td colspan="2">Strengths: This proposal appears to fall in line with the P.I.’s level of development as an investigator. Her mentor is appropriately engaged in the project as part of a strong team to understand and better utilize technology to collect, analyze, and act upon important clinical data related to chronic illness (hypertension) in a more timely and clinically relevant way. Results from this study will provide a strong basis for future external funding.

Weaknesses: This proposed study has been described both as a “feasibility” study as well as a “proof [illegible] concept” trial. A next larger and longer follow-up study may be a true “pilot” study or a smaller scale study as opposed to the RO1 suggested in the proposal.</td></tr>
</table>

Research into Practice (SRC) Scoring Form – Round 18

Similar to that of the NIH, the review process for the Pilot Grant Program (PGP) utilizes the same 9 point scale with no decimals to score individual categories within the five main criteria of Significance, Investigator(s), Innovation, Approach and Environment. **A score of 1 indicates an exceptionally strong application with essentially no weaknesses. A score of 9 indicates an application with serious and substantive weaknesses with very few strengths. Ratings are in whole numbers only (no decimal ratings)**

Overall Impact - The Overall Impact takes into consideration, but is distinct from, the scored review criteria. The reviewer should consider the strengths and weaknesses of the main criteria to determine an overall impact/priority score that reflects their overall evaluation.

* This is NOT a numerical average of individual criterion scores;
* Nor is it necessarily the arithmetic mean of the scores for the scored review criteria.

The Overall Impact paragraph provides the reviewer with the opportunity of explaining how the Overall Impact score was derived (i.e., those factors that contributed to the score). If a project has a strong/weak Overall Impact score then the reviewer should highlight those scored criteria that contributed to the favorable/poor score. For example, if the potential significance of a study was so great as to overshadow a number of methodological weaknesses then this should be clearly stated. Likewise, if the design of the study is so flawed as to negate any potential significance and/or innovation of the study then this should be clearly stated. Importantly, the Overall Impact paragraph should provide a clear justification of those key factors that that led to his/her Overall Impact score. It is not intended to simply summarize and/or restate the strengths and weakness detailed in the critique.

An application does not need to be strong in all categories to be judged likely to have major impact. For example, a project that by its nature is not innovative may be essential to advance a field.

Criterion scores are intended to convey how each assigned reviewer weighed the strengths and weaknesses of each section providing scores without providing comments in the review critique is discouraged.

Each criterion should be assessed based on how important it is to the work being proposed. As a result, a reviewer may give only moderate scores to some of the review criteria but still give a high overall impact/priority score because the one review criterion critically important to the research is rated highly; or a reviewer could give mostly high criterion ratings but rate the overall impact/priority score lower because the

Research into Practice (SRC) Scoring Form – Round 18

one criterion critically important to the research being proposed is not highly rated. A major strength may outweigh many minor and correctable weaknesses.

Score	Guidance on Strengths/Weaknesses	Descriptor	Impact
1	Exceptionally strong with essentially no weaknesses	Exceptional	High
2	Extremely strong with negligible weaknesses	Outstanding	
3	Very strong with only some minor weaknesses	Excellent	
4	Strong but with numerous minor weaknesses	Very Good	Medium
5	Strong but with at least one moderate weakness	Good	
6	Some strengths but also some moderate weaknesses	Satisfactory	
7	Some strengths but with at least one major weakness	Fair	Low
8	A few strengths and a few major weaknesses	Marginal	
9	Very few strengths and numerous major weaknesses	Poor	

**Minor Weakness:** An easily addressable weakness that does not substantially lessen impact
**Moderate Weakness:** A weakness that lessens impact
**Major Weakness:** A weakness that severely limits impact
**Non-numeric score options:**
NRFC = Not Recommended for Further Consideration
DF = Deferred
ND = No Discussion of this submission
