# Supplementary material for: Utilizing Consumer Health Informatics to Support Management of Hypertension by Clinical Pharmacists in Primary Care: Study Protocol
Source: JMIR Res Protoc. 2017 Oct 10;6(10):e193. doi: 10.2196/resprot.8059 (PMC5654738; doi:10.2196/resprot.8059)
Supplement: Multimedia Appendix 2 [file resprot_v6i10e193_app2.pdf]

MICHR MICHIGAN INSTITUTE FOR CLINICAL & HEALTH RESEARCH UNIVERSITY OF MICHIGAN

# Research into Practice (SRC) Scoring Form – Round 18

Project Title: Utilizing Consumer Health Informatics to Support Management of Hypertension

Principal Investigator: Buis

Reviewer 2

| Criteria | Review Questions & Comments | Score |
|---|---|---|
| Overall Impact | **Overall Impact Score:** The likelihood for the project to exert a sustained, powerful influence on the research field(s) involved, in consideration of the following five core review criteria, and additional review criteria (as applicable for the project proposed). Please provide a paragraph summarizing the factors that informed your Overall Impact score. (required) | 5 |
| | Strengths:<br>The PI proposes a feasibility study of a "BYOD" (Bring your own device) approach to remote directed Management of a common chronic health problem by a non-physician care extender, in this case a Clinical pharmacist. The innovation here lies in the potential flexibility and scalability of using patients Own mobile devices to manage a bidirectional link to care. This approach has great potential to Transform care. The study team is very strong. The software at the core of the protocol is promising<br><br>Weaknesses:<br>The study team seems to be lacking needed expertise in qualitative methods, and the description of the qualitative arm of the study is rudimentary and needs more development. It is not clear whether this type of research would be more appropriately pursued as a public-private partnership, sponsored in part by the software vendor. They need to tell what impact this will have at the point of care. Tell us more on how you will evaluate if this is feasible. | |
| Significance | **1.** The potential for this project to address a significant health care challenge and advance mechanistic, diagnostic and /or therapeutic understanding of a clinical problem. If the aims of the project are achieved, how will scientific knowledge, technical capability, and or/clinical practice be improved? How will successful completion of the aims change the concepts, methods, technologies, treatments, services, or preventative interventions that drive this field? **Strengths and Weaknesses** (required) | 2 or 7 |

| | |
|---|---|
| | Strengths:<br>Very common chronic problem, disappointing effectiveness of current interventions.<br>Proposes an extension of an evidence-based intervention supported by at least one large RCT (pharmacist management by telemonitoring) to allow for wider implementation and use of mobile devices already in daily use by patients.<br><br>Weaknesses:<br>There are many apps for this approach, many in field testing now. Is this a line of research that should be supported by MICHR, or by software vendor?<br>Does not discuss or attempt to solve problem of multiple devices/multiple apps for multiple conditions that will be a growing issue, so perhaps less significant ('just one more'). |

MICHR MICHIGAN INSTITUTE FOR CLINICAL & HEALTH RESEARCH UNIVERSITY OF MICHIGAN

# Research into Practice (SRC) Scoring Form – Round 18

<table>
<tr><td rowspan="2">Investigators</td><td>2. Are the PIs, collaborators and other researchers well suited to the project? If Early Stage Investigators, do they have the appropriate experience and training, and is a mentoring program with an established investigator outlined? If the PI is a Senior Investigator (Associate Prof. and above), has it been clearly demonstrated that the proposed work is a departure from prior research? If the project is collaborative or multi-PD(s)/PI(s), do the investigators have complementary and integrated expertise; is their leadership approach, governance and organizational structure appropriate for the project? Strengths and Weaknesses (required)</td><td>3</td></tr>
<tr><td colspan="2">Strengths:<br>Strong team.  Mentor very highly accomplished in this field, PI has excellent preparation and some Prior work to establish credentials and prepare for this project.<br>Pharmacist is strong addition to team, but adding clinical site pharmacist as funded member is the Right call.<br><br>Weaknesses:<br>Unclear whether there is sufficient qualitative research design or qualitative analytic expertise on team.</td></tr>
<tr><td rowspan="2">Innovation</td><td>3. The potential for this project to develop novel concepts, approaches, methodologies, tools or technologies in the field(s). Are the concepts, approaches or methodologies, instrumentation, or interventions novel to one field of research o novel in a broad sense? Strengths and Weaknesses (required)</td><td>4</td></tr>
<tr><td colspan="2">Strengths/weaknesses<br>The potential for small scale health IT to transform practice is tremendous, for good and bad – this is An area full of promise.<br>BUT<br>Innovation oversold by study team.  Innovative Aspects 1 and 2 are not innovative, have already been done, with RCT evidence supporting home BP data to pharmacist and pharmacist management in general.  Aspect 3, the BYOD approach, is the innovation – although this approach is being field tested by vendors now.  Aspect 4, scalability, is hampered by 2 things not addressed in this proposal – the problem of managing multiple apps for multiple conditions (app proliferation) and potential overload of the clinician managing output.  In this preliminary work, neither can be addressed.<br>The innovation is the “interfacing” but that is not captured here.</td></tr>
</table>

| Approach | | |
|---|---|---|
| | **4.** Are the overall strategy, methodology, and analyses well-reasoned and appropriate to accomplish the specific aims of the project? Are potential problems, alternative strategies, and benchmarks for success presented? For research not directly involving humans, has the PI clearly described how the next step in the overall research program will be translated into human-based, clinical research? **Strengths and Weaknesses** | 5 |
| | Strengths:<br>General protocol well-developed, good choice of software, condition, and (short) protocol.<br>Focus on feasibility, rather than hard outcome comparison, is appropriate.<br>Plan to collect data to capture relationship between data collection /clinical decisions / changes in Core outcomes (BP, med changes, adherence) is interesting, but will be difficult to carry off – good Work to include in this pilot study.<br>Interactions between study team and clinic will be smooth.<br><br>Weaknesses:<br>Qualitative aspect of study incompletely described, seems like an afterthought. What questions are Of most interest, to which of potential participants or key stakeholders?<br>What do you want to know about patients' experiences? Pharmacists?<br>Why include physicians, who will likely have little exposure?<br>How many interviews will be done? Sampling strategy?<br>Who will analyze interview data, and how?<br>Presume quantitative data collected to determine feasibility and reliability of the protocol for later clinical trial, since there will be no parallel data collection from patients receiving usual care.<br>12 weeks too short to assess impact on BP, again assume that purpose of these measurements is to Assess feasibility for a more comprehensive, longer study to come. | |

| Environment | | |
|---|---|---|
| | **5.** Will the scientific environment in which the work will be done contribute to the probability of success? Are the institutional support, equipment and other physical resources available to the investigators adequate for the project proposed? Will the project benefit from unique features of the scientific environment, subject populations, or collaborative arrangements? Is the proposed budget and available resources fully justified and adequate to complete the work in the proposed **1 year time period**? **Strengths and Weaknesses** (required) | 1 |
| | Strengths:<br>Team is excellent.<br>Setting (Ypsilanti Fam Med) good, and appropriate for a next-step proposal to test this approach in Underserved population.<br><br>Weaknesses:<br>None. | |

# Research into Practice (SRC) Scoring Form – Round 18

<table>
<tr><td rowspan="2">Program</td><td>6. Overall, does the application meet the objectives of the specific RFA and/or goals of the Pilot Grant Program? Including, but not limited to:<br>➢ To assist early career investigators by providing funding support that will enable them to establish a clinical & translational research path.<br>➢ To assist established basic science investigators to move their research into the translational research arena.<br>➢ To support clinicians interested in pursuing innovative research questions in the clinical setting or in the community.<br>➢ The likelihood that this proposal will lead to external funding.</td><td>3</td></tr>
<tr><td colspan="2">Strengths:<br>PI is well-prepared to do this work, primary mentor excellent and has strong relationship with PI. This arena is rapidly developing, should be fertile over next several years.<br><br>Weaknesses:<br>This is an arena in which private enterprise is moving very fast, much faster than academics. Need to explore public-private partnerships here. Could this proposal have been designed as a partnership with the vendor? Does MICHR need to fund this? No discussion of these opportunities in this proposal.</td></tr>
</table>

# Research into Practice (SRC) Scoring Form – Round 18

Similar to that of the NIH, the review process for the Pilot Grant Program (PGP) utilizes the same 9 point scale with no decimals to score individual categories within the five main criteria of Significance, Investigator(s), Innovation, Approach and Environment. **A score of 1 indicates an exceptionally strong application with essentially no weaknesses. A score of 9 indicates an application with serious and substantive weaknesses with very few strengths. Ratings are in whole numbers only (no decimal ratings)**

**Overall Impact -** The Overall Impact takes into consideration, but is distinct from, the scored review criteria. The reviewer should consider the strengths and weaknesses of the main criteria to determine an overall impact/priority score that reflects their overall evaluation.

* This is NOT a numerical average of individual criterion scores;
* Nor is it necessarily the arithmetic mean of the scores for the scored review criteria.

The Overall Impact paragraph provides the reviewer with the opportunity of explaining how the Overall Impact score was derived (i.e., those factors that contributed to the score). If a project has a strong/weak Overall Impact score then the reviewer should highlight those scored criteria that contributed to the favorable/poor score. For example, if the potential significance of a study was so great as to overshadow a number of methodological weaknesses then this should be clearly stated. Likewise, if the design of the study is so flawed as to negate any potential significance and/or innovation of the study then this should be clearly stated. Importantly, the Overall Impact paragraph should provide a clear justification of those key factors that that led to his/her Overall Impact score. It is not intended to simply summarize and/or restate the strengths and weakness detailed in the critique.

An application does not need to be strong in all categories to be judged likely to have major impact. For example, a project that by its nature is not innovative may be essential to advance a field.

**Criterion scores** are intended to convey how each assigned reviewer weighed the strengths and weaknesses of each section providing scores without providing comments in the review critique is discouraged.

Each criterion should be assessed based on how important it is to the work being proposed. As a result, a reviewer may give only moderate scores to some of the review criteria but still give a high overall impact/priority score because the one review criterion critically important to the research is rated highly; or a reviewer could give mostly high criterion ratings but rate the overall impact/priority score lower because the

one criterion critically important to the research being proposed is not highly rated. A major strength may outweigh many minor and correctable weaknesses.

| Score | Guidance on Strengths/Weaknesses | Descriptor | Impact |
|---|---|---|---|
| 1 | Exceptionally strong with essentially no weaknesses | Exceptional | High |
| 2 | Extremely strong with negligible weaknesses | Outstanding | |
| 3 | Very strong with only some minor weaknesses | Excellent | |
| 4 | Strong but with numerous minor weaknesses | Very Good | Medium |
| 5 | Strong but with at least one moderate weakness | Good | |
| 6 | Some strengths but also some moderate weaknesses | Satisfactory | |
| 7 | Some strengths but with at least one major weakness | Fair | Low |
| 8 | A few strengths and a few major weaknesses | Marginal | |
| 9 | Very few strengths and numerous major weaknesses | Poor | |

| |
|---|
| **Minor Weakness:** An easily addressable weakness that does not substantially lessen impact |
| **Moderate Weakness:** A weakness that lessens impact |
| **Major Weakness:** A weakness that severely limits impact |
| |
| **Non-numeric score options:** |
| NRFC = Not Recommended for Further Consideration |
| DF = Deferred |
| ND = No Discussion of this submission |
